# Reassessing diagnostic accuracy claims in case only study of T-shaped uterus

**DOI:** 10.1007/s00404-026-08319-0

**Published:** 2026-01-30

**Authors:** Onur Ince, Gonca Ozten Dere, Ali Can Gunes, Lale Karakoc Sokmensuer

**Affiliations:** 1 Department of Obstetrics and Gynecology, Faculty of Medicine, Hacettepe University, Ankara, Turkey; 2 Department of Histology and Embryology, Faculty of Medicine, Hacettepe University, Ankara, Turkey

Monaco et al. [1] compared three-dimensional transvaginal ultrasound (3D-TVS) morphometric classifications for T-shaped uterus proposed by Exacoustos et al. [2], Ludwin et al. [3] (Classification of Uterine Morphological Evaluation – CUME, 2020), and Pacheco et al. [4] (“Rule of 10”) and synthesized new combinations of their criteria that, according to their analysis, yielded the best diagnostic performance with very high receiver-operating-characteristic/area-under-the-curve (ROC/AUC) values. However, these results are not analytically valid due to the dataset composition, study design, and statistical methodology.

The most fundamental issue is that ROC analysis requires both positive and negative cases, as it describes how sensitivity changes relative to 1 – specificity. The dataset included only confirmed T-shaped cases (*n* = 50) without any normal or non-dysmorphic uteri, which makes the use of ROC/AUC statistically invalid. Specificity can only be calculated when true negatives exist. Clinically, this is like testing a pregnancy test only on pregnant women: it shows how many are correctly detected, but says nothing about how often healthy non-pregnant women would be mislabeled as pregnant. Any AUC derived from a dataset containing only positive cases, therefore, does not measure diagnostic ability; it merely reflects how consistently the tested criteria overlap within already diagnosed cases. Thus, the criteria combinations presented by the authors do not demonstrate diagnostic validity.

The pattern of reported results—where lateral bulging (LB) ≥ 5 mm outperforms LB ≥ 7 mm, lateral indentation angle (AI) ≤ 140° outperforms AI ≤ 130°, “ ≥ 3 of 3” criteria are outperformed by “ ≥ 2 of 3,” and even “ ≥ 2 of 3” are outperformed by “ ≥ 1 of 3”—is entirely predictable in a case-only design. Broader thresholds inevitably classify a larger proportion of the same positive cohort as positive, yielding higher apparent AUCs, since the analysis never penalizes false positives. In other words, the broader the rule, the better it will appear to perform under these conditions. Indeed, if no threshold were used at all—or if criteria so broad as “LB ≥ 0 mm” or “AI ≤ 360°” were applied—labeling all 50 cases as T-shaped would yield a perfect AUC. This occurs because the calculations implicitly assume that all parameters are 100% specific (evident from the reported AUC values) and would never classify a healthy uterus as T-shaped, which is misleading. The methodology, therefore, identifies not truly accurate diagnostic markers but progressively more inclusive rules that assign the T-shaped label to a larger fraction of a preselected positive sample.

A further issue is circular validation. Although the diagnoses were ultimately confirmed by hysteroscopy, the cases selected for hysteroscopy were first diagnosed as T-shaped by sonographers. Those sonographers’ subjective judgments determined which cases were included as “true T-shaped,” and they may have relied on the same criteria later evaluated in the study. Consequently, the morphometric parameters tested later were not independent of the diagnostic process that determined inclusion. The analysis may thus be rediscovering the experts’ own decision patterns rather than identifying new, independent predictors of the condition. If the sonographers indeed used these or similar measurements in their assessments, the study essentially measures how well the sonographers’ preferred classification systems align with their own impressions—or which system they most frequently employ—rather than how accurately those systems diagnose the anomaly. This methodological circularity, which conflates derivation and validation within the same decision framework, should have been clearly recognized and discussed as a limitation.

The multivariate logistic regression analysis is also unclear. Logistic regression requires a dichotomous outcome variable, but only T-shaped uteri were analyzed. Unless the dependent variable represented a different internal classification (for example, meeting certain criteria versus not meeting them within the T-shaped cohort), the regression is mis-specified. A proper predictive model for T-shaped versus non-T-shaped morphology would require a comparison group of normal and other dysmorphic uteri measured with the same morphometric parameters. The scope and intent of this regression should therefore have been detailed.

Overall, the real strength of the work lies in codifying morphometric descriptors and promoting standardization among examiners, which can facilitate better communication and reproducibility in future research. Nevertheless, the findings cannot be interpreted as evidence of diagnostic accuracy. The proposed cut-offs should be tested prospectively in a dataset that includes both normal and variant uterine morphologies, with independent assessment and proper estimates of sensitivity, specificity, and predictive values. Until such validation is performed, the reported measurements define how experts agree on what they already recognize as T-shaped, rather than how accurately T-shaped uteri can be diagnosed in general clinical practice. A genuine diagnostic study would require a control group, blinded classification, and evaluation of whether these morphometric features correlate with relevant clinical outcomes such as fertility rates or benefit from hysteroscopic metroplasty.

## Data Availability

No datasets were generated or analysed during the current study.
